# Mechanisms of Central Hypogonadism

**DOI:** 10.3390/ijms22158217

**Published:** 2021-07-30

**Authors:** Thomas M. Barber, Ioannis Kyrou, Gregory Kaltsas, Ashley B. Grossman, Harpal S Randeva, Martin O. Weickert

**Affiliations:** 1 Warwickshire Institute for the Study of Diabetes, Endocrinology and Metabolism, University Hospitals Coventry and Warwickshire, Clifford Bridge Road, Coventry CV2 2DX, UK; kyrouj@gmail.com (I.K.); harpal.randeva@uhcw.nhs.uk (H.S.R.); 2 Division of Biomedical Sciences, Warwick Medical School, University of Warwick, Coventry CV2 2DX, UK; 3 Aston Medical Research Institute, Aston Medical School, College of Health and Life Sciences, Aston University, Birmingham B4 7ET, UK; 4 Centre for Sport, Exercise and Life Sciences, Faculty of Health & Life Sciences, Coventry University, Coventry CV2 2DX, UK; 5 National and Kapodistrian University of Athens, 10679 Athens, Greece; gkaltsas@endo.gr; 6 Green Templeton College, University of Oxford, Oxford OX2 6HG, UK; ashley.grossman@ocdem.ox.ac.uk; 7 Barts and the London School of Medicine, University of London, London E1 2AD, UK

**Keywords:** hypogonadism, prolactin, leptin, Kallmann syndrome, stress

## Abstract

Reproductive function depends upon an operational hypothalamo–pituitary–gonadal (HPG) axis. Due to its role in determining survival versus reproductive strategies, the HPG axis is vulnerable to a diverse plethora of signals that ultimately manifest with Central Hypogonadism (CH) in all its many guises. Acquired CH can result from any pituitary or hypothalamic lesion, including its treatment (such as surgical resection and/or radiotherapy). The HPG axis is particularly sensitive to the suppressive effects of hyperprolactinaemia that can occur for many reasons, including prolactinomas, and as a side effect of certain drug therapies. Physiologically, prolactin (combined with the suppressive effects of autonomic neural signals from suckling) plays a key role in suppressing the gonadal axis and establishing temporary CH during lactation. Leptin is a further key endocrine regulator of the HPG axis. During starvation, hypoleptinaemia (from diminished fat stores) results in activation of hypothalamic agouti-related peptide neurons that have a dual purpose to enhance appetite (important for survival) and concomitantly suppresses GnRH neurons via effects on neural kisspeptin release. Obesity is associated with hyperleptinaemia and leptin resistance that may also suppress the HPG axis. The suppressibility of the HPG axis also leaves it vulnerable to the effects of external signals that include morphine, anabolic-androgenic steroids, physical trauma and stress, all of which are relatively common causes of CH. Finally, the HPG axis is susceptible to congenital malformations, with reports of mutations within >50 genes that manifest with congenital CH, including Kallmann Syndrome associated with hyposmia or anosmia (reduction or loss of the sense of smell due to the closely associated migration of GnRH with olfactory neurons during embryogenesis). Analogous to the HPG axis itself, patients with CH are often vulnerable, and their clinical management requires both sensitivity and empathy.

## 1. Introduction

Unlike other physiological and endocrine systems, survival is unshackled from the function of the reproductive (hypothalamo–pituitary–gonadal (HPG)) axis. This unique independence of the reproductive axis from survival prospects, positions the HPG axis as an essential switch between survival and reproductive modes, a switch influenced by many signals from diverse origins, and one that underlies the origin of central hypogonadism (CH) in its many guises and clinical scenarios. Accordingly, the HPG axis in both sexes manifests exquisite sensitivity to a great plethora of signals, undergoes suppression relatively easily, and acts as the body’s early warning system or “harbinger” of disease or distress. From an evolutionary perspective, it is perhaps not surprising that such a scenario exists. Many functions within the endocrine system (such as the hypothalamo–pituitary–adrenal (HPA) [1] and thyroid axes [2] that regulate cortisol and thyroid hormone production, respectively) form an essential and vital role for survival. Accordingly, the functioning of such vital systems is typically robust, and is maintained despite the co-occurrence of disease and distress (albeit with enhanced release of cortisol [3,4] and sometimes the sick euthyroid syndrome [5]). Conversely, the HPG axis does not directly fulfil a vital role in survival. Indeed, such independence of the HPG axis from a direct survival function occurs naturally post-menopause [6]. Furthermore, reproduction is a facultative process. In times of disease or distress, there is targeting of resources towards survival mode, and a shift away from reproductive strategies [7]. Put simply, when survival is threatened, there is usually suppression of the HPG axis and therefore diminished reproductive functioning until such threats have receded.

Hypogonadism refers to a reduction or absence of sex hormone secretion or other reproductive activity of the testes or ovaries. Hypogonadism is classified both clinically and biochemically as primary hypogonadism (PH), reflecting defects at the level of the gonadal tissue, and CH, reflecting defects within the central hypothalamo-pituitary reproductive unit [8,9]. Biochemically, hypogonadism manifests with low serum levels of the sex steroids (testosterone and oestradiol in men and women, respectively), and variable serum levels of the gonadotrophins, Luteinising hormone (LH) and Follicle stimulating hormone (FSH), according to the level of the defect [8]. The clinical manifestations of hypogonadism include subfertility, reproductive dysfunction (anovulation and amenorrhoea in women; erectile dysfunction and non-obstructive azoospermia in men [10]), and non-specific features such as reduced vitality and wellbeing, physical weakness, bone mineral loss, and to some extent emotional lability and reduced libido [11,12]. The vulnerability of the HPG axis renders CH as a relatively common clinical scenario, especially in the context of obesity (predominantly in males) and severe stress [13]. Indeed, it seems likely that CH is underdiagnosed, given its frequent clinical presentation with non-specific features. Furthermore, it seems likely that there is reduced awareness of CH as a clinical entity among non-specialist healthcare professionals.

Although the causes of CH incorporate both congenital and acquired factors, its essential feature is a lack of gonadal stimulation from the hypothalamo-pituitary axis [14]. Although CH underlies infertility in only 1–2% of male cases [15], these patients usually respond relatively well to fertility treatments [10]. Acquired causes of CH mostly develop during or after puberty, and can result from any type of pituitary [16,17] or hypothalamic lesion (including prolactinomas and other pituitary neoplasms [18], craniopharyngiomas and hypothalamic tumours [19]), and/or iatrogenically from the treatments of such lesions (including surgical resection and radiotherapy) [20]. Further acquired causes of CH include pituitary trauma, the use of exogenous steroids, pituitary iron overload from hereditary haemochromatosis or beta-thalassemia, and infiltrative conditions including hypothalamic disorders [14,21,22]. Ultimately, these conditions of acquired CH impair the function of gonadotroph cells within the anterior pituitary and/or reduce the function of gonadotrophin releasing hormone (GnRH) neurons, and thereby reduce the release of FSH and LH with consequent impaired spermatogenesis and testicular dysfunction in males, and an absence of menstruation and hence sub-fertility in females [14,23]. The severity of CH is commensurate with the severity of the underlying condition [21]. Congenital CH is synonymous with idiopathic central hypogonadism (ICH), an umbrella term to classify the failure of embryonic migration of GnRH neurons into the hypothalamus [21].

**Figure 1:**
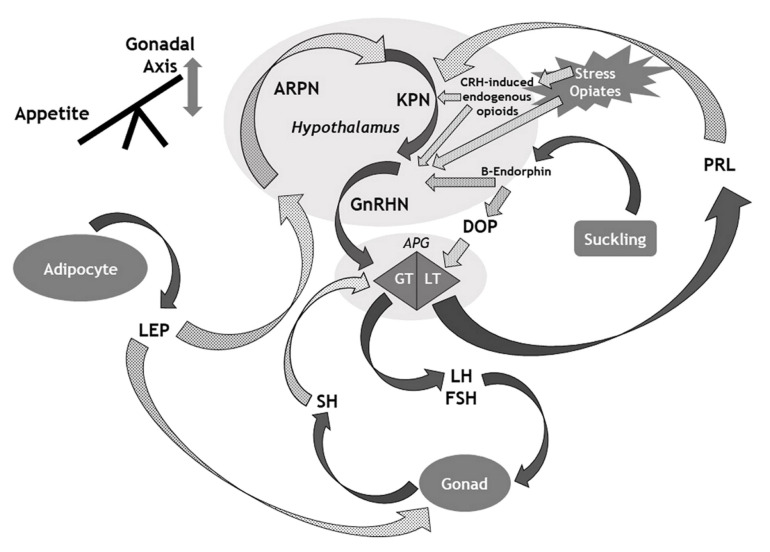
Overview of the main mechanisms that influence the gonadal axis. APG: Anterior pituitary gland; ARPN: Agouti-related peptide neurons; CRH: Corticotrophin-releasing hormone; DOP: Dopamine; FSH: Follicle stimulating hormone; GnRHN: Gonadotrophin releasing hormone neurons; GT: Gonadotroph cells; KPN: Kisspeptin neurons; LEP: Leptin; LH: Luteinising hormone; LT: Lactotroph cells; PRL: Prolactin; SH: Sex hormone. Solid arrows denote stimulatory effects (or hormonal release); Dotted (grey) arrows denote suppressive effects.

Here, we provide a narrative overview of the key mechanisms involved in CH. We illustrate this with an overview of the genetics of ICH and implications for our understanding of the proper functioning and ontogeny of the HPG axis. We then discuss the numerous mechanisms whereby multiple signals from diverse origins impact on the HPG axis (illustrated in Figure 1) to provide insight into the physiological functioning of the HPG axis, and the numerous mechanisms that underlie the development of CH.

## 2. Methods

We performed a narrative literature review using PubMed. We used the search term “central hypogonadism”, and prioritised published papers from the last 10 years (although not exclusively), to enable modern insights from the latest published data.

## 3. The HPG Axis: Lessons from the Genetics of Idiopathic Central Hypogonadism (ICH)

Considerable insight has been gained into the ontogeny, development and functioning of the HPG axis through studying patients with genetic mutations that result in ICH. The numerous identified gene mutations for ICH fall naturally into three main groups that control: (i) neuronal differentiation and migration, (ii) GnRH secretion and (iii) the development of GnRH receptors [21,24]. Each gene mutation has a variable effect on GnRH neuronal migration or functioning that in turn determines the severity of the clinical phenotype. This ranges from mild constitutional delay of puberty to more severe phenotypes. It is beyond the scope of this concise review to provide a detailed exposition of the genetics of ICH, recently outlined elsewhere [25]. Rather, we provide a summary of the salient points, and develop this as a clinical guide to provide insight into the normal development and functioning of the HPG axis, and the mechanisms that underlie the development of CH.

There is subdivision of ICH into Kallmann Syndrome (KS) with the co-presence of olfactory dysfunction (anosmia or hyposmia), and normosmic ICH, although these sub-divided entities likely sit on a spectrum within ICH as a whole [21,26]. KS is a genetically heterogeneous disorder and underlies much CH globally [21,27]. In addition to anosmia or hyposmia, patients with KS may also present with cleft lip and palate, sensorineural deficits, motor impairment and renal agenesis [21]. The majority of hypothalamic GnRH neurons have their origin within the olfactory placode at around the 5th week of gestation [25]. These GnRH neurons migrate alongside vomeronasal, olfactory and terminal nerves to reach their destination in the infundibulum, mediobasal hypothalamus and periventricular region [25,28]. The shared origin of nerves for olfaction and GnRH provides an explanation for the association of ICH with olfactory impairment in around 50% of cases of ICH [25,29]. Although *ANOS1* mutations in hemizygosity nearly always associate with a complete loss of olfaction, those gene variants implicated in GnRH neuron migration and axon guidance, and neurodevelopmental genes involved in the specification and differentiation of GnRH neurons, are associated with a range of olfactory defects [25]. Due to their independence from the co-migration of olfactory and GnRH neurons, gene variants that impair the action of GnRH at the gonadotroph level, or are implicated in the activation of GnRH neurons or the secretion of GnRH, do not associate with olfactory defects [25,30].

There is heterogeneity in the transmission of gene mutations for ICH that include sporadic (the majority), autosomal dominant, autosomal recessive and X-linked inheritance [21,31]. Overall, approximately half of cases of ICH have identified mutations within around 50 genes, of which around 20 genes associate with KS [32]. Many of the genes implicated in the development of ICH encode for G-protein-coupled receptors and (less frequently) their cognate ligands [25]. *ANOS1* (previously known as *KAL1*) was the first gene identified with mutations that associate with KS [33], and occurs in ~10% of men with KS (although implicated in 33–70% of familial cases) [21,34]. Mutations within *ANOS1* transmit through familial X-linkage and influence early migration of GnRH neurons [35]. Conversely, mutations within *FGFR1* (affecting ~12% of men with KS [36]) transmit through an autosomal dominant mode, and together with mutations in *PROK1* and *PROK2* (G-protein coupled receptors) influence both GnRH neuronal migration and olfactory bulb development [37,38]. Other genes implicated in the pathogenesis of KS include *GNRHR* that encodes the GnRH receptor [39], *CHD7* [40] and *FGF8* [41]. Patients harbouring deleterious variants of *FGF8* have a greater prevalence of other congenital anomalies that include dental agenesis, midline facial defects and digital bone abnormalities [25,42]. Furthermore, sensorineural hearing loss occurs in 5–15% of patients with ICH (with a higher prevalence in KS) [25,43], and is associated with mutations in *ANOS1*, *SOX10* and *CHD7* [25,44].

In a subset of patients, KS co-exists with other neuroendocrine disorders. It is likely that the gene mutations that underlie these disorders influence common pathways early in the process of embryogenesis, with multiple clinical manifestations [21,45]. Examples of such gene mutations include those within *SOX10* (KS in the context of Waardenburg-Shah syndrome) [46], *HESX1* (KS in the context of pituitary hormone deficiency and septo-optic dysplasia) [47], *NROB1* (ICH in the context of congenital adrenal dysplasia) [45] and *LEP* (ICH in the context of obesity) [45].

Although substantial progress has been made in recent years regarding insights into the genetic basis of ICH, various aspects remain uninvestigated, with apparent variability in the penetrance and expressivity of gene variants that probably reflects oligogenicity of gene variants (both known and unknown) [25]. Certain deleterious gene variants within *FGFR1*, *TUBB3* and *CHD7* for example, may manifest with ICH in the heterozygous state [25]. Furthermore, in cases of homozygosity and compound heterozygosity in ICH, there are commonly genotype–phenotype correlations. However, for many cases of gene variants that underlie ICH, correlations between genotype and phenotype remain uncertain, and it can be difficult to disentangle the relative effects of known and unknown genetic variants [25]. Although there has been a transformation of our knowledge and understanding of the genetic aetiology of ICH in recent years, unfortunately the complexity of these mechanisms outlined here limit the clinical utility and benefits of effective genetic counselling in many cases of ICH.

## 4. Prolactin as a Natural Contraceptive

As outlined in the last section, ICH incorporates a whole spectrum of genetic anomalies that provides insight into the ontogeny and functioning of the HPG axis, and through dysfunction of GnRH neuronal regulation, the development of CH. In this section, we consider the role of prolactin in the regulation of the HPG axis, and the mechanisms whereby raised serum prolactin can result in the development of CH.

For many decades, there has been recognition of lactation as a natural contraceptive, and a means of gaining central control of reproductive function [48]. Indeed globally, the “lactational amenorrhoea method” remains important for contraception and family planning [49]. During suckling, afferent neural inputs pass from the nipple to the hypothalamus via the spinal cord. These signals cause a local release of beta-endorphin that results in suppression of GnRH secretion, with consequent suppression of gonadotrophin release and arrested ovarian follicular development, ovulation and menstruation [49]. To complement the direct suppression of GnRH secretion, the hypothalamic release of beta-endorphin also suppresses the production of dopamine that in turn results in increased production and secretion of prolactin from the anterior pituitary gland [49]. The mechanism(s) by which raised serum prolactin mediates the suppression of gonadotrophin release during lactation and suckling remains incompletely understood. However, recent evidence demonstrates that prolactin inhibits the hypothalamic secretion of kisspeptin. The suppression of kisspeptin release in turn results in reduced stimulation of GnRH release and development of CH [50], a process which also involves endogenous endorphin or dynorphin [51]. It is likely that kisspeptin neurons also contribute towards the feedback regulation of prolactin secretion [50].

In women, lactation represents a natural form of CH, mediated through the release of hypothalamic beta-endorphin with consequent disruption of the HPG axis through both direct and indirect mechanisms as outlined, the latter implicating the release of prolactin from the anterior pituitary gland. This is teleologically of value as it prevents further conception while the infant is fully reliant on breast milk. However, raised serum prolactin can occur outside of the context of lactation in both men and women, and exert suppressive effects on the HPG axis with the induction of CH. Prolactin is one of the apparent stress hormones. During stressful situations, serum levels of prolactin may become elevated [52]. The direct inhibitory effect of raised prolactin on the HPG axis may ensure optimal conditions for pregnancy through its avoidance during times of maternal stress. However, it remains unclear as to why amenorrhoea persists later in lactation when serum prolactin is only minimally elevated.

Raised serum prolactin can also occur following the development of a prolactinoma [53]. Clinically, prolactinomas often present with features of CH in both men and women. However, compared with women, men with prolactinomas usually present clinically with larger tumour size and a greater elevation of serum prolactin [54]. A likely explanation for this sex difference in the clinical presentation of prolactinoma is that amenorrhoea serves as an early warning signal to women (particularly those with a history of regular periods) that often prompts early medical attention. In men, there is no equivalent to a monthly menstrual period, and therefore no aberration to any cyclical marker of HPG functionality; in men, it is difficult to know when the reproductive “clock” stops ticking. Conversely, prolactinoma-related reduced libido and erections will not necessarily prompt early medical attention by men, and may go unnoticed or attributed to stress for example, or even ignored due to embarrassment [55]. Consequently, men with prolactinomas typically present late compared with women, and may even have developed some of the tumour compressive features associated with pituitary macroadenomas, including bitemporal hemianopia [56]. However, as microprolactinomas so rarely develop into macroadenomas, this is unlikely to be the sole reason.

In addition to prolactinomas, any hypothalamo-pituitary lesion (including non-functioning pituitary tumours and craniopharyngiomas) can be associated with the development of CH [17]. There are likely multiple mechanisms implicated that include the suppression of gonadotrophin release from the tumour itself, and “disconnection hyperprolactinaemia” [57]. There are suppressive effects of dopamine release on the pituitary lactotroph cells, following its transit down the pituitary stalk [58]. Therefore, any lesion that impinges on the pituitary stalk has the potential to disrupt dopamine signalling to the lactotroph cells, manifesting with hyperprolactinaemia and thus a loss of stimulatory GnRH effect with consequent CH. Furthermore, loss of pituitary stalk connectivity may directly impair stimulation of gonadotrophin release and hypothalamic GnRH function. During the clinical evaluation of hyperprolactinaemia, disconnection hyperprolactinaemia is generally associated with a serum level of prolactin <2000 mIU/L [59], although rarely it may be higher.

A further clinically important aetiology of raised serum prolactin (and potential CH) includes that related to drug side-effects (summarised in Table 1). During the clinical assessment of patients with hyperprolactinaemia, it is essential to elicit a careful drug history to exclude any potential side effects that may adversely affect the HPG axis and induce CH. Most commonly, neuroleptic drug therapies associate with hyperprolactinaemia. These include the classical anti-psychotic therapies (phenothiazines, butyrophenones and thioxanthenes) [60], and some atypical antipsychotic therapies such as risperidone [61]. Indeed, with chronic usage of these anti-psychotic therapies, 40–90% of patients experience elevated serum levels of prolactin, with clinical manifestations of amenorrhoea, galactorrhoea and erectile dysfunction [61,62]. A number of the newer anti-depressant therapies can also induce hyperprolactinaemia. These include selective serotonin reuptake inhibitors, serotonin and noradrenaline reuptake inhibitors [61,63] and tricyclic antidepressants [61,64]. The prokinetic drugs metoclopramide and domperidone (through potent dopamine antagonism) also commonly induce hyperprolactinaemia in a majority of patients taking these therapies [61,65,66]. Finally, other drugs implicated in raised serum prolactin include methyldopa [67], verapamil [68], opiates [69], alcohol, cocaine and marijuana [61,66]. Unlike patients with prolactinomas (with levels of serum prolactin typically between 2000 and 5000 mIU/L for microprolactinoma, and >10,000 mIU/L for macroprolactinoma) [53], patients with hyperprolactinaemia secondary to drug therapy typically only have mild elevations of serum prolactin (usually, but not invariably, <2000 mIU/L). Following identification of a potential drug-induced cause of hyperprolactinaemia, discontinuation of the suspected drug usually results in normalisation of serum prolactin levels and restoration of a normally functioning HPG axis. Following discontinuation of oral antipsychotic drug therapy, serum prolactin levels typically fall into the normal range within 2 to 4 days [61,70]. Persistent hyperprolactinaemia should prompt a search for alternative causes.

## 5. Leptin as a Gatekeeper between Survival and Reproduction

From an evolutionary perspective, both lactation and stress are potent suppressors of the HPG axis. While the mediated influence of lactation on HPG axis suppression occurs through the effects of prolactin release from the anterior pituitary, most forms of stress suppress the HPG axis through mechanisms that are independent of an elevated serum prolactin. However, other than stress, in addition to prolactin, leptin represents an important determinant of HPG functioning in both men and women. Leptin acts within a relatively narrow range of serum concentrations, any deviation from which can result in compromise of HPG functioning [71]. In this section, we discuss the variable effects of leptin on the HPG axis, acting as a gatekeeper according to its level within the serum.

### 5.1. Euleptinaemia and Hypoleptinaemia

Leptin is an adipokine released from adipose tissue depots commensurately with the volume of adipose tissue [72]. As such, serum leptin serves as a useful biomarker of adipose tissue reserve and provides an important peripheral signal to the hypothalamo-pituitary unit that controls the HPG axis [73]. When adequate fat stores exist, providing a propitious scenario for any putative pregnancy, serum levels of leptin are within the normal range (euleptinaemia), and leptin acts as a hormone of permission [74]. In this scenario of adequate fat reserves, leptin allows normal functioning of the reproductive axis to ensue.

As the main hormone that communicates stored energy status to the rest of the body, leptin has pleiotropic effects on numerous endocrine and neuronal systems. This includes regulation of anterior pituitary hormones such as growth hormone, prolactin and adrenocorticotrophic hormone (ACTH) [75]. However, leptin influences reproductive function indirectly. Indeed, neurons that express GnRH do not actually express the leptin receptor (OB-R) [76]. Rather, leptin acts on neurons within the hypothalamic ventral pre-mamillary nucleus that express a dense concentration of leptin receptors [71], and that project to hypothalamic regions that control reproductive function [77]. Leptin plays a key role in appetite regulation and suppresses activity within the orexigenic agouti-related peptide neurons. Agouti-related peptide suppresses the activity of kisspeptin neurons that in turn reduces activity within GnRH neurons. Therefore, in euleptinaemia, suppression of agouti-related peptide enables the release of GnRH and normal functioning of the HPG axis. Conversely, in times of starvation when fat reserves are low, with consequent reduction in the serum levels of leptin (hypoleptinaemia), there is activation of agouti-related peptide neurons that in turn suppress activity within kisspeptin cells with consequent suppression of GnRH release, reproductive function and leads to the development of CH [71]. Evidence from a rodent-based study showed the mediation of the effects of leptin on the HPG axis (including the kisspeptin system), at least in part, through effects on the nitrinergic system, especially within the arcuate nucleus [76]. The nitric oxide-containing cells surround the GnRH neurons in a basket-like arrangement [78].

To summarise, leptin acts indirectly to control the activity of GnRH neurons (and therefore reproductive functioning), via effects on the activity of kisspeptin neurons. Pathways upstream of kisspeptin neuron regulation implicate agouti-related peptide neurons and the nitrinergic system [76]. Accordingly, the central control of the HPG axis functions like a switch between survival and reproductive modes of physiology, with levels of leptin as a major determinant of its status. Survival always takes precedence over reproduction. As such, the reproductive axis is particularly sensitive to any signalling of survival threat, particularly starvation from a relative paucity of food availability and/or intake. Mediated through effects on diminished serum levels of leptin from depleted fat stores, any chronic energy deficiency can suppress the HPG axis. This includes anorexia nervosa, lipodystrophies and relative energy deficiency in the context of sports, malnutrition and starvation [79]. In one cross-sectional study of elite athletes and dancers, it was shown that changes in both serum cortisol and insulin-like growth factor binding protein 1 (IGFBP-1) accounted for the majority of the variance in menstrual cyclicity [80]. As an independent predictor of amenorrhoea, IGFBP-1 may act as a peripheral signal to the central control of the HPG axis, and mediate information on the availability of metabolic fuel for the control of reproductive function (given the direct inverse regulation of IGFBP-1 levels by serum insulin) [80]. In addition to excessive exercise, weight loss resulting from any chronic and/or systemic illness (including malignancies and infectious diseases) can also result in suppression of the HPG axis [79].

Appetite control is regulated within the hypothalamus, with agouti-related peptide as the main orexigenic signal, and pro-opiomelanocortin (POMC) as a key anorexigenic signal. It is interesting that in the context of hypoleptinaemia, the hypothalamic release of agouti-related peptide (and concomitant reduced stimulation of hypothalamic POMC release) appears to have a dual purpose to enhance appetite for food and suppress reproductive function. Therefore, in the constant interplay and balance between survival (in this case, the avoidance of starvation) and reproduction, agouti-related peptide appears to play a central role.

### 5.2. Hyperleptinaemia

Weight gain with accumulation of excess adipose tissue and obesity associate with elevated serum levels of leptin (hyperleptinaemia) [81,82]. Analogous to the context of hypoleptinaemia, the development of hyperleptinaemia also results in suppression of the HPG axis with resultant CH. However, unlike hypoleptinaemia, the mechanisms that mediate the effects of hyperleptinaemia on suppression of the HPG axis remain incompletely understood [83]. Obesity, particularly in the context of type 2 diabetes (T2D), is related to the development of resistance to the effects of leptin. Effectively, obesity-associated leptin resistance may have similar effects to hypoleptinaemia on the HPG axis, with central suppression of GnRH secretion mediated through enhanced hypothalamic release of agouti-related peptide [84]. In addition to central suppression of the HPG axis, hyperleptinaemia also exerts direct negative effects on LH and hCG-stimulated testicular androgen production and reduces the responsiveness of the testicular Leydig cells to gonadotrophin stimulation [85]. Therefore, obesity-related hyperleptinaemia may suppress the HPG axis at multiple levels both centrally and, at least in men, peripherally through a reduced testicular response to gonadotrophins.

In male obesity, especially in the context of type 2 diabetes (T2D), numerous other factors contribute towards the development of CH that include enhanced conversion of testosterone to oestradiol within adipose tissue from enhanced aromatase activity [86], and consequent central suppressive effects of oestradiol on the HPG axis. Furthermore, insulin resistance, hyperinsulinaemia and associated inflammatory mediators may also suppress HPG functioning [82]. One such inflammatory mediator, Interleukin-1β (IL-1β), is a potent downregulator of the HPG axis during immune-inflammatory challenge in both men and women [87]. IL-1β suppresses the hypothalamic secretion of GnRH, with consequent suppression of LH release from the anterior pituitary gland, and the development of CH. In one study on explants of anterior pituitary gland from ewes, IL-1β also had direct effects on pituitary gonadotroph cells, with suppression of GnRH-stimulated LH release and *LH*-β gene expression [87]. Therefore, IL-1β may mediate the association of inflammatory conditions (including the chronic inflammatory milieu of obesity) with suppressive effects on the HPG axis [87]. In one study on men with features of the metabolic syndrome, there was a significant inverse correlation between serum interleukin-6 (IL-6) and testosterone levels [88]. Therefore, in addition to IL-1β, IL-6 probably also plays an important role in the mediation of the suppressive effects of visceral obesity (and associated low-grade inflammation) on the male HPG axis [88]. Finally, it is important to emphasise that in both men and women, generalised inflammation and cytokine release likely contribute towards the suppression of the HPG axis, even outside of the context of obesity and the metabolic syndrome. For example, changes in inflammatory markers and serum testosterone are likely causally linked in ageing men [89] and in patients with cancer-related cachexia [90].

To summarise this section, leptin appears to play a central role as a gatekeeper between survival and reproductive modes of physiology. For optimal reproductive functioning, there needs to be an ideal metabolic status that allows for euleptinaemia (serum leptin levels within a narrow range). Chronic restriction of food intake with diminished fat stores associates with hypoleptinaemia that switches off the reproductive axis, and concomitantly enhances appetite for food (an important component of survival mode). Conversely, chronic caloric excess with expanded adipose tissue depots associates with hyperleptinaemia and leptin resistance, the reproductive and appetitive effects of which are similar to that which occurs in the context of hypoleptinaemia due to diminished effectiveness of leptin on its receptor, combined with the peripheral suppressive effects of hyperleptinaemia on the testicular response to gonadotrophins. In this way, appetite for food and reproductive functioning behave like two ends of a seesaw, with leptin and agouti-related peptide at its fulcrum.

## 6. Signals from the Outside: Morphine, Anabolic-Androgenic Steroids, Physical Trauma and Stress

The HPG axis is vulnerable to signals from diverse origins. Having explored the endocrine contributors to HPG functioning (including the roles of prolactin and leptin), in this section we consider the impact of external signals on the HPG axis. Numerous environmental signals influence the proper functioning of the HPG axis. By definition such signals originate from outside of the body. Furthermore, these signals have diverse modalities that include both chemical and physical insults. We illustrate the mechanisms by which external signals disrupt the HPG axis through the examples of morphine, anabolic-androgenic steroids (AAS), physical trauma and stress.

### 6.1. Morphine

Human usage of opium dates back 8000 years. During the Renaissance, the creation of various opium products resulted in common usage and subsequent addiction [91]. Morphine, the active ingredient of opium, was isolated chemically in the early 1800s by Wilhelm Sertürner, with subsequent elucidation of its chemical and structural formula by Sir Robert Robinson in the 1940s [91]. Plant-based extraction of morphine from the poppy plant remains a major source of morphine-based therapies [91]. Currently, there is a stark disparity and inequality in the global usage of opiates, with 80% of the world opiate supply consumed by the United States (US) and 80% of the global population having no access to opiates [92]. There was a sharp increase in opiate prescriptions and deaths from opiate overuse in the 2000s, culminating in a global opiate crisis [92]. In a review of the literature, Rose identified a peak in opiate analgesic prescribing and opiate overdose deaths in 2011, with subsequent long-term decline [92]. There was a subsequent sharp increase in opiate overdose in 2014, driven by illicit use of heroin and fentanyl. Currently, such illicit heroin and fentanyl usage (rather than opiate prescribing) fuels the opiate crisis [92]. However, the widespread use of oxycodone, driven by pharma support, is also a major contributor to the current opiate crisis.

The negative effects of opiates are legion, and their effects on male reproduction are well known [93]. However, there seems to be a general lack of awareness among healthcare professionals regarding the potential for CH in male users of opiates, and this discussion is often absent during clinically-based treatments with opiate therapies, including those discussions with patients [93]. In a recent systematic review and meta-analysis of opiate effects on the HPG axis, de Vries and colleagues identified 52 studies from the literature, with >18,400 subjects [94]. Indications for opiate usage (such as methadone and morphine) included chronic pain and maintenance treatment for opiate addiction. Overall, the prevalence of opiate-related CH was 63% (the vast majority, 99.5% being male subjects), and opiate-related hypocortisolism occurred in 15% of subjects [94]. The authors concluded that patients on chronic opiate therapy should undergo periodic evaluation of the HPG and HPA axes [94]. In addition to the development of CH, exogenous opiates also reduce semen quality and increase DNA fragmentation [93].

Although opiates exert their negative effects on the HPG axis through a variety of mechanisms, their major mechanism of action is via the direct inhibition of GnRH release [95,96]. Opiate-related increases in prolactin release can also suppress gonadotrophin production and secretion from the anterior pituitary gland [93]. There are also direct negative effects of opiates on the testes, even in the context of well-maintained levels of androgens. Endogenous production of opioids (within testicular Leydig and germ cells) exert their paracrine effects through interaction with receptors throughout the testes (including the regulation of androgen binding protein from effects on Sertoli cell receptors) [93]. There are also effects of opioids on the expression of aromatase within the brain and testis [93] that may further suppress the proper functioning of the HPG axis.

Given the prevalence of CH in male chronic users of opiates, with the majority having some degree of CH, it seems surprising that this very common and potentially debilitating side-effect of opiates is not more widely appreciated and discussed with male patients prior to and at the time of prescription of opiate-based therapies. It is incumbent upon healthcare professionals, especially those who prescribe opiates, to discuss the potential (indeed likelihood in male patients) for CH development with patients, and the clinical features of CH and the known adverse effects on testicular function and male fertility. Furthermore, proper endocrine assessment of HPG functioning both prior to initiation of opiates, and periodically whilst on opiates, is important to enable the early identification and appropriate and timely management of opiate-related CH.

### 6.2. Anabolic-Androgenic Steroids (AAS)

AAS are testosterone derivatives typically used either recreationally or by athletes to enhance strength and appearance and/or to improve athletic performance [97]. The use of AAS is a relatively common cause of CH in clinical practice, the global lifetime prevalence of AAS use being 6.4% and 1.6% for men and women, respectively [98]. However, despite the common usage of AAS, there is a limitation in our understanding of the adverse effects of AAS and the mechanism(s) implicated through a lack of controlled clinical trials in this field due to ethical constraints [98]. Therefore, much of our understanding derives from retrospective cohort studies and case reports. Such published data reveal that the use and withdrawal of AAS commonly causes CH that may be profound in its severity and frequently prolonged, and may cause substantial morbidity, including potentially irreversible adverse effects on the HPG axis [97,99]. Rahnema and colleagues conducted a review of the relevant literature and showed that symptoms of hypogonadism in men who use AAS depends on the dose, duration and type of AAS used [100]. In one systematic review and meta-analysis reported by Christou and colleagues, based on >3800 primarily male subjects (>1700 AAS users) from >30 reported studies, most AAS users had hypogonadism with persistently low serum levels of testosterone and gonadotrophins lasting several weeks to months following discontinuation of AAS [97]. Furthermore, AAS associated with functional and structural changes in sperm, subfertility, gynaecomastia and reduced testicular volume in men, and in women, clitoromegaly and menstrual irregularities [97]. In a further study on weight-lifters from the US, compared with non-AAS users those who had formerly used AAS and had not been treated showed significantly smaller testicular volumes and lower levels of serum testosterone that persisted in some cases for >2 years [99]. Furthermore, this untreated group of former AAS users had significantly lower scores of sexual desire, and in the group of former AAS users (both treated and untreated), 29% had experienced major depressive episodes during the withdrawal of AAS [99].

The mechanisms of CH associated with the use of AAS stems from negative feedback in the central regulation of the HPG axis [98]. In women who use AAS, such HPG axis dysregulation may manifest with disruption of ovarian function, acne, hirsutism and deepening of the voice that may be irreversible [98]. Indeed, the clinical features of AAS in women may masquerade as both the female athlete triad (menstrual disorders, low bone mass and low energy intake) [98] and Polycystic Ovary Syndrome. This highlights the importance of a careful history and examination in women who present with such common clinical features to exclude any potential for use of AAS.

In addition to the adverse effects on the HPG axis, the use of AAS also associates with cognitive and brain abnormalities similar to those in Alzheimer’s Disease. Supra-physiological levels of androgens within the brain resulting from usage of AAS induce androgenic abnormalities and oxidative stress that associate with changes in the expression and activity of proteins that synthesize and eliminate β-amyloid and hyperphosphorylated tau protein, with resultant accumulation of these abnormal proteins within the brain [101]. Such accumulation may accelerate through the concomitant usage of other psychoactive substances, ultimately leading to an increased risk of the early development of dementia [101].

### 6.3. Physical Trauma

The hypothalamo-pituitary unit is particularly vulnerable to any physical trauma to the head. Factors that may contribute towards this vulnerability include its anatomical position at the base of the brain, and the portal circulation of the anterior pituitary, enhancing its risk of injury from infarction following any trauma-related compromise to its blood supply. Traumatic Brain Injury (TBI) is a relatively common cause of CH, most commonly affecting younger adults under the age of 35 years. Pituitary dysfunction (including CH and other pituitary deficits such as hypothyroidism, hypocortisolism, growth hormone deficiency and central diabetes insipidus) occurs in 20–40% of patients following moderate and severe TBI in both the acute and chronic phases [102]. Although the pathogenesis of hypopituitarism (including CH) following TBI remains incompletely understood, hypotheses include direct injury from the primary mechanical event itself, and secondary effects that include hypoxia, hypotension, changes in cerebral blood flow and metabolism and raised intracranial pressure, that may ultimately induce ischaemic adenohypophysial infarction [102]. In addition to acute TBI, even relatively minor but repeated head injuries (such as through boxing, martial arts and football (“heading’ the ball”)) can cause impairment of HPG function and the development of CH [103,104,105,106,107].

### 6.4. Stress

The term “stress” has multiple definitions across diverse fields. Even within the clinical realm, “stress” is used as an umbrella term with multiple meanings. In this context, we use the term “stress” to indicate a state of mental or emotional strain or tension resulting from adverse or demanding circumstances. Stress often originates from the outside. Furthermore, “adverse or demanding circumstances” are entirely subjective and likely to affect different people in different ways [108,109]. Any change can have potential for eliciting a stress response, even if personally perceived as having positive utility. We should not assume therefore that stress only occurs following negative life events. Accordingly, stress within our busy, demanding, dynamic, unpredictable and highly changeable modern-day world, is very common [110].

It is well established that stress can induce CH [111]. The clinical implications of stress-induced CH include the development of amenorrhoea in girls, for example, in the context of stress from examinations, and the onset of erectile dysfunction in men who experience stress. As the existing literature on stress-induced CH appears orientated towards women, we focus our discussion on “Stress-Amenorrhoea” (SA). In women, SA is common and is thought to account for 15–48% of menstrual disorders [112,113]. Ultimately, SA ensues from activation of multiple neural, metabolic and hormonal pathways that culminate in a reduction of the pulsatile secretion of GnRH from hypothalamic neurons [111,112]. During acute and chronic stress, there is activation of the sympathetic adreno-medullary and HPA axes, respectively [114]. In addition to activation of the HPA axis, stress-related activation of corticotrophin-releasing hormone (CRH) may also enhance the release of endogenous opioid peptides and endorphins that in turn impair the pulsatile release of GnRH [112,115]. This is a mechanism of CH similar to the use of exogenous opiates [95]. Other mechanisms that underlie SA include the impairment of kisspeptin-secreting neurons [112]. Interestingly, although prolactin may be considered as a “stress hormone”, SA is probably not mediated by hyperprolactinaemia. Indeed conversely, SA may associate with low levels of serum prolactin due to stress-induced dopaminergic overtone [112,116]. Finally, we should generally consider SA as a diagnosis of exclusion [112].

To summarise this section, the vulnerability of the HPG axis stems from its evolutionary susceptibility to and necessity for suppressibility. Perhaps as a side-effect of the hyper-responsiveness of the HPG axis to multiple internal (including neuronal and endocrine) signals, the HPG axis is also vulnerable to signals from the outside, such as the use of opiates, AAS, physical trauma and stress. In one sense, the over-accommodating nature of the HPG axis has left it vulnerable to being “gate-crashed” by unwanted guests.

## 7. To Treat or Not to Treat?

In any clinical scenario, the potential for CH exists. It is important to appreciate that the identification of CH does not necessarily imply an urgent need for treatment with hormone replacements [117]. Indeed, during acute illness or distress, the temporary occurrence of CH may actually fulfil a protective role. It is apposite to consider the occurrence of CH in its clinical scenario, including physiological CH such as during lactation or menopause. In certain scenarios, such as CH from underweight and hypoleptinaemia, a clinical decision is required regarding the need for hormone replacement. However, in these scenarios, it is likely that hormone replacements are used for their non-reproductive benefits (such as bone [11] and cardiovascular protection [118]), and may require adequate contraception if pregnancy is considered harmful. A detailed discussion of treatment and management options for CH is beyond the scope of this review and has been covered extensively recently in an Endocrine Society Clinical Practice Guideline [117]. However, given the diversity and complexity of the many clinical scenarios that manifest in CH, it is important to consider each case individually. Furthermore, future research should explore options to complement the administration of sex hormone replacement therapies for the treatment of CH, such as the use of taurine [119].

Although the HPG axis controls reproductive function, it is important to consider the non-reproductive effects of CH. As noted above, CH over the longer term can adversely affect bone [11] and cardiovascular health [118], and result in reduced muscle mass and physical strength [120]. Furthermore, CH can manifest with metabolic dysfunction [121], reduced vitality, irritability and changes in cognition and emotional functioning. It is important to raise awareness of these non-reproductive features of CH to ensure early institution of appropriate investigations and diagnosis, and to enable timely referral to an endocrine specialist. In women, the sudden loss of menstrual periods with the onset of CH serves as an early warning. Unfortunately, there is no cyclical reproductive equivalent in men. Therefore, CH can be more insidious in men, and may go unnoticed, or the non-reproductive features of CH attributed to something else such as stress. It is important therefore, particularly in men and especially in the context of risk factors, to have a low threshold for investigating CH and for prompt specialist referral when required. Thorough investigation and management of CH should include consideration of its associated sexual and reproductive dysfunctions with attendant emotional ramifications, and the non-reproductive features of CH. Analogous to the HPG axis itself, the patient with CH is often vulnerable.

## 8. Conclusions

To conclude, the HPG axis is vulnerable to numerous factors that include endocrine inputs (effects of prolactin, leptin and β-endorphin); neural signals (such as occur during suckling); inflammatory molecules (including interleukins and cytokines); stress; weight-loss and energy-deficient states; hypothalamo-pituitary lesions (including disconnection hyperprolactinaemia); and numerous external signals such as opiates, AAS, physical trauma and stress. The complex ontogeny of the GnRH neurons with diverse genetic regulation further enhances the vulnerability of the normal function of the HPG axis. Finally, numerous drug therapies (outlined in Table 1) are associated with raised serum prolactin with potential for the development of CH.

The facultative nature of the reproductive mode stems from a need to conserve resources for survival in times of need, and to avoid the untimely allocation of resources for reproduction at such times, that would further threaten survival. Indeed, in one reported study on >21,600 couples married between 1860 and 1895 from the Utah Population Database, increased number of offspring and reproductive rate actually correlated with reduced parental survival, this correlation being significantly more pronounced for mothers than for fathers [122]. Unsurprisingly, parental mortality resulted in reduced survival and reproductive success of offspring [122]. These data highlight the fitness and survival costs of reproduction within a human population, albeit from the 19th Century. Given the implications of reproduction for survival, it is evolutionarily important to ensure appropriate modes of survival versus reproduction at any given time. Accordingly, the regulation of the HPG axis involves multiple inputs from a diverse array of origins, all of which impact on its proper functioning. Ultimately, the HPG axis functions like a simple switch: “off” for survival mode (denoting CH), and “on” for reproductive mode (denoting normal reproductive functioning).

Finally, there is an important unmet clinical need for raised awareness of CH and its numerous and varied causes amongst healthcare professionals. We need to ensure that we always position the patient centrally in our clinical approach to CH, and to execute our management both sensitively and with empathy.

## Figures and Tables

**Table 1:** Summary of drug causes of hyperprolactinaemia.

Drug Group	Drug Class	Drug Name
Neuroleptic	Classical anti-psychotic	Phenothiazines
		Butyrophenones
		Thioxanthenes
	Atypical anti-psychotic	Risperidone
Anti-depressant	Selective Serotonin Reuptake Inhibitor	Sertraline, Citalopram, Fluoxetine, Paroxetine
	Serotonin and Noradrenaline Reuptake Inhibitor	Duloxetine, Venlafaxine
	Tricyclic antidepressant	Amitriptyline, Nortriptyline
Anti-emetic	Prokinetic	Metoclopramide
		Domperidone
Anti-hypertensive	Centrally-acting alpha-2 adrenergic agonist	Methyldopa
Anti-arrhythmic and Anti-hypertensive	Calcium-Channel Blocker	Verapamil
Depressant	Analgesic	Opiates
	Recreational and “social” drug	Alcohol
Stimulant	Illicit drug	Cocaine
Depressant, Stimulant and Hallucinogen	Recreational drug	Marijuana

## Data Availability

Not applicable.
